# Low-concentration iron promotes *Klebsiella pneumoniae* biofilm formation by suppressing succinic acid

**DOI:** 10.1186/s12866-022-02518-w

**Published:** 2022-04-11

**Authors:** Kexin Liu, Shuang Tan, Weiyuan Ye, Limin Hou, Binghu Fang

**Affiliations:** 1 National Risk Assessment Laboratory for Antimicrobial Resistance of Animal Original Bacteria, South China Agricultural University, Guangzhou, China; 2 Guangdong Provincial Key Laboratory of Veterinary Pharmaceutics Development and Safety Evaluation, South China Agricultural University, Guangzhou, China

**Keywords:** *Klebsiella pneumoniae*, Biofilm, Metabolomics, Fe^2 +^, Succinic acid

## Abstract

**Background:**

*Klebsiella pneumoniae* is widely distributed in water and plays a major role in both human and poultry infections. Many *K. pneumoniae* strains form biofilms on various surfaces, enhancing their pathogenicity and resistance to antibiotics. The water supply pipeline of chicken farms has become a hotbed for the growth of *K pneumoniae* biofilm because of its humid environment, and because the chicken drinking water pipeline is thin, it is easily blocked by the biofilm, and the diffused cells can cause repeated and persistent infections. Iron is vital to the growth of microorganisms and the formation of biofilms. Therefore, the aim of this study was to examine the effects of iron on *K. pneumoniae* biofilm formation and any associated metabolic changes to provide a rationale for reducing the formation of biofilms.

**Results:**

Biofilm formation was enhanced to the greatest extent by the presence of 0.16 mM FeCl_2_, producing a denser structure under electron microscopy. The number of biofilm-forming and planktonic bacteria did not change, but protein and polysaccharide concentrations in the bacterial extracellular polymeric substances (EPS) were significantly increased by iron supplementation. To clarify this mechanism, intracellular metabolomic analysis was carried out, showing that the differential, down-regulated metabolites included succinic acid. The addition of 1.7 mM succinic acid counteracted the biofilm-forming effect of iron, with no bactericidal side effects.

**Conclusion:**

This study demonstrates the importance of succinic acid and iron in *K. pneumoniae* biofilms, and provides insight into the formation of *K. pneumoniae* biofilms and direction for the development of new antibacterial agents.

## Background

Biofilms are dynamic and structurally complex microbial communities that develop on biotic and abiotic surfaces [1, 2]. In nutrient-rich aquatic ecosystems most bacteria develop into biofilms adhering to a substrate [3]. Bacteria fixed in such biofilms differ significantly from planktonic bacteria and are enriched by sub-populations of persister cells that are dormant variants and highly tolerant of antibiotics [4]. Biofilms render antibiotics less effectively by restricting the penetration of the antibiotic, slowing bacterial growth, or altering gene expression [5]. It is, therefore, vital to find strategies to prevent the development of biofilms. *K. pneumoniae* is a Gram-negative bacillus found in human lungs and intestines and is a major pathogen associated with both hospital and community-acquired infections causing lung tissue necrosis and abscesses [6]. Unfortunately, the carbapenemase-producing *K. pneumoniae* discovered at the beginning of this century is resistant to almost all known antibiotics [7, 8]. *K. pneumoniae* can form biofilms on various medical materials including lung cannulae, cardiac pacemakers and other internal devices, which undoubtedly enhances its pathogenicity and drug resistance [9].

Iron is an essential element for bacterial growth, participating in various processes such as the tricarboxylic acid cycle, oxygen and electron transport, and the respiratory chain. It is also a cofactor for a variety of microbial proteins and enzymes that are essential for biofilm formation [10, 11]. In bacteria, iron regulates surface motility and promotes biofilm formation by stabilizing the polysaccharide matrix [12]. Cells acquire appropriate quantities of key metals at the start of the shift from primary to secondary metabolism. These essential micronutrients regulate the expression of genes responsible for synthesis of secondary metabolites and/or morphological alterations associated with cellular differentiation [13]. The differentiation of planktonic cells into sessile cells is associated with many stress factors and environmental factors [14]. Groundwater, the source of drinking water for animals, contains iron, so water supply pipelines are a natural hotbed of biofilm growth due to the presence of moist surfaces and key metals [15]. Poultry drinking water pipelines in particular are thin and susceptible to blockages caused by biofilms. Furthermore, individual cells and multicellular clusters can be released, facilitating systemic dissemination and the infection of secondary sites [16, 17]. *K. pneumoniae* infection is a major cause of death in chicken embryos and two-week-old chicks, and omphalitis with high morbidity and mortality. Epidemiological studies have shown that Klebsiella can survive for a long time in chicken coops, appliances, and the environment, significantly impacting infections in poultry and humans [18, 19].

This study samples drinking water from a chicken farm and explores the effect of iron on *K. pneumoniae* biofilm formation and associated metabolic changes, providing insight and direction for research into biofilm inhibition.

## Materials and methods

### Chemicals

Crystal violet, N-acetyl-L-glutamic acid, succinic acid and anthrone were purchased from Shanghai Macklin Biochemical Technology Co., Ltd. (Shanghai, China). Luria-Bertani (LB) agar, LB broth and MacConkey agar were purchased from Guangdong Huankai Microbial Sci. & Tech. Co., Ltd. (Guangdong, China). Glycerin, pure anhydrous glucose, NaCl, FeCl_2_, and ethylenediaminetetraacetic acid disodium salt (EDTA-2Na) were purchased from Shanghai Yuanye Bio-Technology Co., Ltd. (Shanghai, China). Methanol, formic acid, and ammonium acetate (HPLC grade) were purchased from Thermo Fisher Scientific (Waltham, MA, USA). Phosphate buffered saline and concentrated sulfuric acid were purchased from Shanghai Runjie Chemical Reagent Co., Ltd. (Shanghai, China).

### Bacterial strains and growth conditions

Forty-nine strains of *K. pneumoniae* were isolated from samples collected from a medium-sized chicken farm located in Yunfu City, Guangdong Province, China*.* Thirty-four strains were isolated from the upstream and downstream water system and the remaining 15 from biofilm scraped from the wall of the water supply pipeline. All strains were stored in 30% glycerol Luria broth (LB) at − 80 °C. Before use, strains were rejuvenated by freshly plating from frozen tubes, inoculating into LB broth, and cultivating at 37 °C. Bacteria were then inoculated onto LB agar medium, cultured at 37 °C, single colonies inoculated into LB and subcultured for further use.

### Biofilm formation microtiter plate assay

*K. pneumoniae* was grown in LB at 37 °C until the concentration of the bacterial suspension reached 0.5 McDonnell’s turbidity. The culture was diluted 100-fold to a concentration of 10^6^ colony-forming units (CFU)/mL, placed in 96-well plates (200 μL/well, eight wells per strain) and incubated statically at 37 °C for 24 h. The biofilm formation assay was performed essentially as described by O’Toole & Kolter [20]. Planktonic cells were then removed and wells were washed three times with phosphate buffered saline (PBS; pH 7.2–7.4) to remove unattached cells. Methanol (200 μL, 99%) was added to each well and fixed for 20 min. After discarding the methanol, plates were dried at room temperature. Attached cells were stained for 30 min with 200 μL of 0.5% crystal violet. Unbound dye was removed by rinsing with copious running water. Plates were dried upside down in a hot-air oven. Ethanol (200 μL) was added to each well to dissolve the bound dye and the absorbance was recorded at 590 nm as a measure of biofilm formation (mean of eight wells).

### Biofilm formation with iron supplementation

To explore the effects of iron on biofilms, one strong film-forming bacterial strain (YT-9) was selected. Microtiter plate wells were inoculated with 200 μL of *K. pneumoniae* YT-9 culture equivalent to a bacterial cell density of 10^6^ CFU/ml along with various concentrations of FeCl_2_ (eight replicates containing 0, 0.016, 0.16, 1.6, and 16 mM). Plates were incubated at 37 °C for 24, 48, 72, and 96 h and biofilm content were determined using the crystal violet staining method as above. The optimum iron concentration and cultivation time for biofilm growth was then applied to the other 48 strains to determine if these conditions were universal for all *K. pneumoniae*.

To determine the number of viable bacteria under optimal culture conditions, biofilms were grown in 96-well microtiter plates (eight replicates). Culture liquid (100 μL) was removed from each well, added to 900 μL sterile NaCl, serially diluted, and 20 μL plated on LB agar. Plates were incubated at 37 °C and CFU counted after 6 h. Unattached bacteria were discarded, each well was washed three times with PBS, the biofilm on the well sides and bottom were wiped with sterile cotton swabs, the swabs were placed in 30 mL saline, sonicated for 15 min, and vortexed for 1 min. Bacterial suspension (100 μL) was mixed with 900 μL sterilized NaCl and CFU were counted as above.

### Development of biofilm on PVC (polyvinyl chloride)

Changes in the structure of biofilms under low-concentration iron conditions were examined using Erlenmeyer flasks. Each flask contained 150 mL LB and 1.5 mL of bacterial liquid at 0.5 McFar’s turbidity (giving a concentration of 10^6^ CFU/mL) and the optimum concentration of iron. A PVC plastic sheet (2 × 5 cm) was added to each flask as a surface for biofilm attachment, simulating the substrate of a poultry drinking water pipe. The sheets were previously washed with dishwashing detergent and 75% ethanol to remove bacteria and grease, then rinsed with sterile ultrapure water and dried. The culture flasks were incubated at 37 °C for 24 h. PVC sheets were removed using tweezers, unattached bacteria were rinsed off with sterile water, and the sheets were fixed in 99% methanol for 20 min. Sheets were then rinsed with sterile water and oven dried before cutting into small pieces. Bacterial distribution and biofilm structure were observed by electron microscopy.

### EPS extraction and quantitative analyses

To determine changes in extracellular polymeric substances (EPS), biofilms were grown in 96-well microtiter plates. The main components of EPS secreted by biofilm bacteria are proteins and polysaccharides. The EDTA method was used to extract EPS. This method has a low lysis rate for bacteria and high extraction efficiency for both proteins and polysaccharides [21, 22]. Similar to the CFU counting method above, biofilm in the wells was wiped with a cotton swab, placed in 5 mL sterile ultrapure water, and sonicated for 15 min. Pre-cooled (4 °C) 2% EDTA (5 mL) was added, vortexed for 1 min, incubated at 4 °C for 3 h, then centrifuged at 5000 r/min for 30 min. The supernatant was filtered through a 0.22 μm membrane and stored at − 20 °C before use.

Protein content was determined using a BCA kit. Bicinchoninic acid (BCA) and Cu^2+^ solutions were mixed at a ratio of 50:1 (working solution) and a 0.5 mg/mL protein solution was used as the standard. To 200 μL working solution in 2 mL Eppendorf tubes were added 4 μL of distilled water, standard protein solution, or test solution (blank, standard, and sample tubes, respectively). After mixing, tubes were incubated in a 60 °C water bath for 30 min, then 200 μL was transferred to a 96-well plate and absorbance (A) was measured at 562 nm using a microplate reader (A1, A2, and A3, respectively). Protein concentration (C) in EPS was calculated as follows:

$$\mathrm{C}\ \left(\mathrm{mg}/\mathrm{mL}\right)=\mathrm{standard}\ \mathrm{concentration}\ \mathrm{x}\ \left(\mathrm{A}3-\mathrm{A}1\right)/\left(\mathrm{A}2-\mathrm{A}1\right)$$

The anthrone colorimetric method were used for determination of polysaccharides. Pure anhydrous glucose (100 mg) was dissolved in 100 mL deionized water and diluted to 100 μg/mL. Anthrone (0.1 g) was dissolved in 100 mL 80% concentrated sulfuric acid to prepare a 0.1% anthrone reagent. To generate a standard curve, glucose solution (0, 0.2, 0.4, 0.6, 0.8, and 1 mL) was placed in clean, dry glass test tubes, made up to 1 mL with deionized water, and 5 mL anthrone reagent was added. Rubber stoppers were inserted and tubes immediately placed in an ice bath. Once cooled, tubes were placed in a boiling water bath for 10 min then cooled again in the ice bath before transferring 200 μL to a 96-well plate and measuring the absorbance at 620 nm. OD620 of the standards minus the blank control was plotted against glucose concentration and a regression equation generated. EPS extracts (1 mL) were mixed with 5 mL anthrone reagent, treated as above, and the polysaccharide content determined from the standard curve.

### Metabolomic analysis

To explore the mechanism by which iron affects the formation of biofilms, differences between the intracellular non-targeted metabolisms of the control and experimental groups were measured under optimal conditions. The planktonic bacteria in the control and treatment groups were centrifuged at 5000 r/min for 5 min at 4 °C, the supernatant was discarded, and the bacterial pellet was rinsed three times with pre-cooled (4 °C) PBS. The cells were transferred to a 2 mL Eppendorf tube and snap-frozen with liquid nitrogen to halt their metabolism. Eight replicates were prepared from each group and stored at − 80 °*C. prior* to metabolomic analysis, samples were melted on ice and 300 μL of 80% methanol (aq.) was added before refreezing in liquid nitrogen for 5 min. After melting again, samples were vortexed for 30 s, ultrasonicated for 6 min and centrifuged at 5000 r/min for 1 min at 4 °C. The supernatant was transferred to a clean Eppendorf tube, freeze dried to a dry powder, and dissolved in 100 μL 10% methanol solution before analysis by LC-MS/MS.

A Vanquish UHPLC system (Thermo Fisher, Germany) coupled with a Q Exactive HF-X Quadrupole-Orbitrap Hybrid Mass Spectrometer (Thermo Fisher, Germany) was used for sample analysis. Chromatographic separation was performed on a Hypersil Gold C18 column (100 × 2.1 mm, 1.9 μm; Thermo Fisher, Germany) at 40 °C. Gradient elution was performed at a flow rate of 0.2 mL/min with mobile phases consisting of (A) 0.1% formic acid in water and (B) methanol for positive ion mode, and (A) 5 mM ammonium acetate and (B) methanol for negative ion mode (Table 1). The scan range of the mass spectrum was m/z 100–1500. Electrospray ionization (ESI) was used in positive and negative ion modes. Quality Control (QC) samples were included in the sample queue to monitor and evaluate the stability of the system to ensure the reliability of the data. Raw data were preprocessed using CD3.1 data processing software. Data were screened by retention time, mass-to-charge ratio, and other parameters. Sample peak alignment was performed according to retention time deviation and mass deviation (parts per million, ppm) to improve identification accuracy. Peak areas were then quantified, taking into account the set ppm, signal-to-noise ratio (S/N), ion adducts and other information for peak extraction. Metabolites were identified using the high-resolution secondary spectrogram databases mzCloud and mzVault and the MassList primary database (search library). The identified metabolites are functionally and taxonomically annotated, and major databases include Kyoto Encyclopedia of Genes and Genomes (KEGG), Human Metabolome Database (HMDB), LIPID MAPS, etc. Use these databases to annotate the identified metabolites to understand the functional properties and classification of different metabolites; KEGG contains multiple databases, of which the KEGG PATHWAY database is a collection of metabolic pathways. Pathway analysis can determine the most important biochemical metabolic pathways and signal transduction pathways involved in metabolites, using unsupervised principal component analysis (PCA) and supervised partial least squares discriminant analysis (PLS-DA) methods to screen for differential metabolites. Hierarchical cluster analysis was used to assess the metabolic patterns of the metabolites under different experimental conditions. Metabolites with similar patterns may have similar functions or participate in the same metabolic processes or cellular pathways. KEGG is a powerful tool for in vivo metabolic analysis and metabolic network research. Pathway enrichment can identify the most important biochemical metabolic pathways and signal transduction pathways involved in differential metabolites. Let N be the number of metabolites involved in the KEGG metabolic pathway in all metabolites, n be the number of differential metabolites in N, y be the number of metabolites annotated to one KEGG pathway, and x be the number of differential metabolites enriched in this KEGG pathway. If the ratio condition x / n > y / N is satisfied, the pathway is KEGG enrichment pathway. The hypergeometric test method was used to obtain the *P*-value of pathway enrichment, where P-value ≤0.05 was taken as the threshold. The KEGG pathway meeting this condition was defined as the KEGG pathway that was significantly enriched in differential metabolites. The enrichment results are displayed with a KEGG enrich scatterplot.

**Table 1:** UHPLC mobile phase gradient

Time (min)	Flow rate (mL/min)	A %	B %
0.0	0.2	98	2
1.5	0.2	98	2
12.0	0.2	0	100
14.0	0.2	0	100
14.1	0.2	98	2
17.0	0.2	98	2

### Metabolism verification

To verify the impact of various metabolites on biofilm formation, functional tests were performed on the down-regulated metabolites identified by KEGG analysis. A series of concentration gradients of different metabolites was applied to create a control group, an iron treatment group, a metabolite group, and a metabolite and iron interaction group. The crystal violet semi-quantitative staining method was used to determine biofilm forming ability. The number of planktonic and film-forming bacteria and changes in EPS were monitored as described above.

### Statistical analysis

All tests were performed with at least eight replicate analyses and repeated three times on different days. Two-tailed Student’s *t*-test was used to identify differences between control and experimental groups, differences being considered significant when *P* was < 0.05. Data were analyzed using Excel and GraphPad Prism 8 software and reported as mean ± one standard deviation.

## Results

### Low concentration iron promotes biofilm formation

**Fig. 1:**
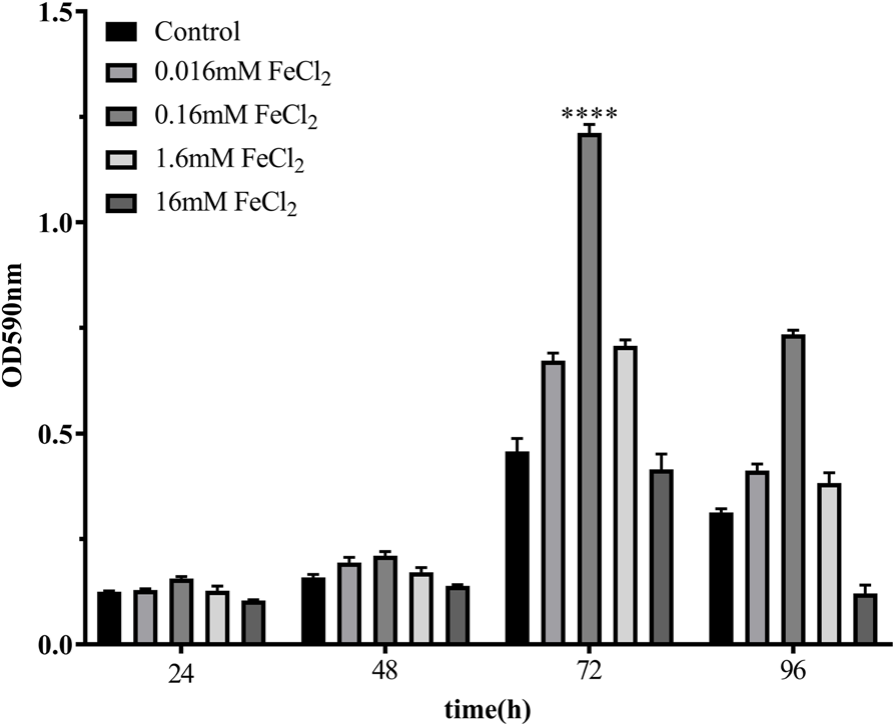
Biofilm formation by *K. pneumoniae* YT-9 in minimal media with and without FeCl_2_ supplementation_._^****^P < 0.0001 (0.16 mM FeCl_2_ vs. control group at 72 h)

**Fig. 2:**
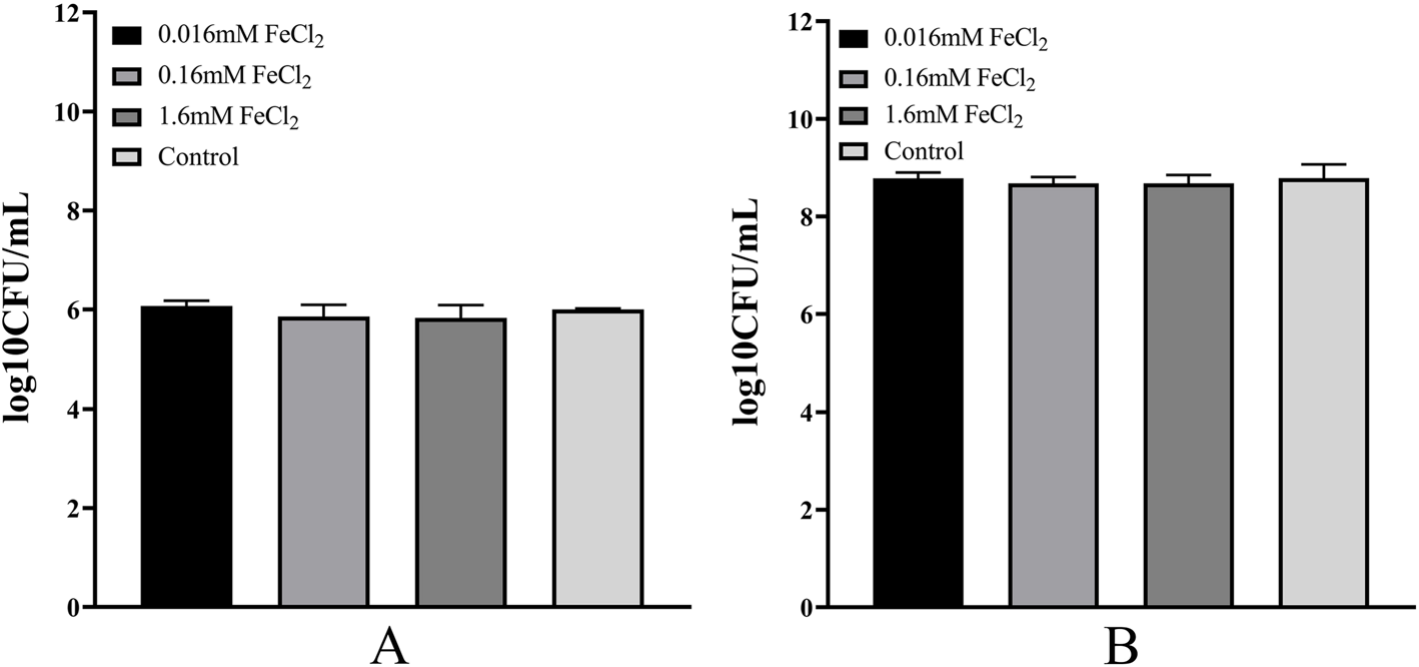
Number of biofilm-forming bacteria (**A**) and planktonic bacteria (**B**) at different FeCl_2_ concentrations of *K. pneumoniae* YT-9

The effect of supplementation with different concentrations of FeCl_2_ on biofilm growth was studied in microtiter plates using *K. pneumoniae* YT-9. Adding 0.16 mM FeCl_2_ and culturing for 72 h consistently promoted biofilm formation compared with the control group without iron (*P* < 0.0001). Supplementation with 0.016 mM and 1.6 mM FeCl_2_ promoted biofilm formation to a lesser degree. Biofilm formation peaked at 72 h under all conditions (Fig. 1). There were no significant differences in the numbers of planktonic and biofilm-forming bacteria at different FeCl_2_ concentrations (Fig. 2). Biofilm formation by the other 48 *K. pneumoniae* strains (cultured for 72 h with 0.16 mM FeCl_2_) varied compared with the control group but was consistently lower than the YT-9 strain.

### Effects of iron supplementation on biofilm structure and EPS

**Fig. 3:**
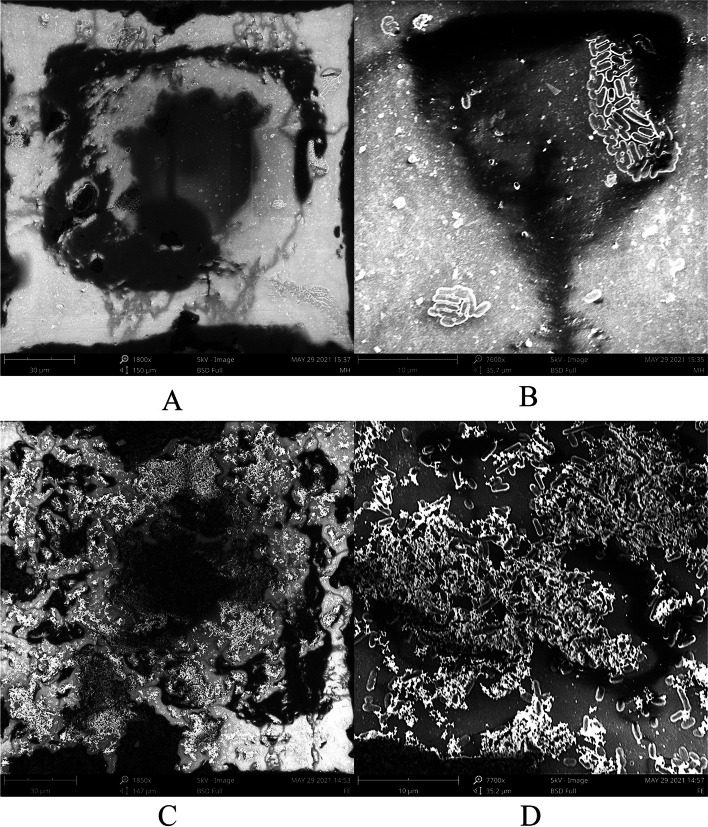
*K. pneumoniae* YT-9 biofilm structure on PVC sheets in the control group (**A, B**) and the 0.16 mM iron-supplemented group (**C, D**) at low (30 μm) and high (10 μm) magnification.

The formation of biofilms on PVC sheets was studied to simulate the substrate for biofilm growth in drinking water pipelines on chicken farms. Electron microscopy revealed that biofilm in the control group as shown in Fig. 3A (at 30 μm) and Fig. 3B (at 10 μm) was thin and bacteria exist in clusters. While the iron-supplemented group as shown in Fig. 3C (at 30 μm) and Fig. 3D (at 10 μm), the biofilm was denser and the bacteria were more evenly distributed.

**Fig. 4:**
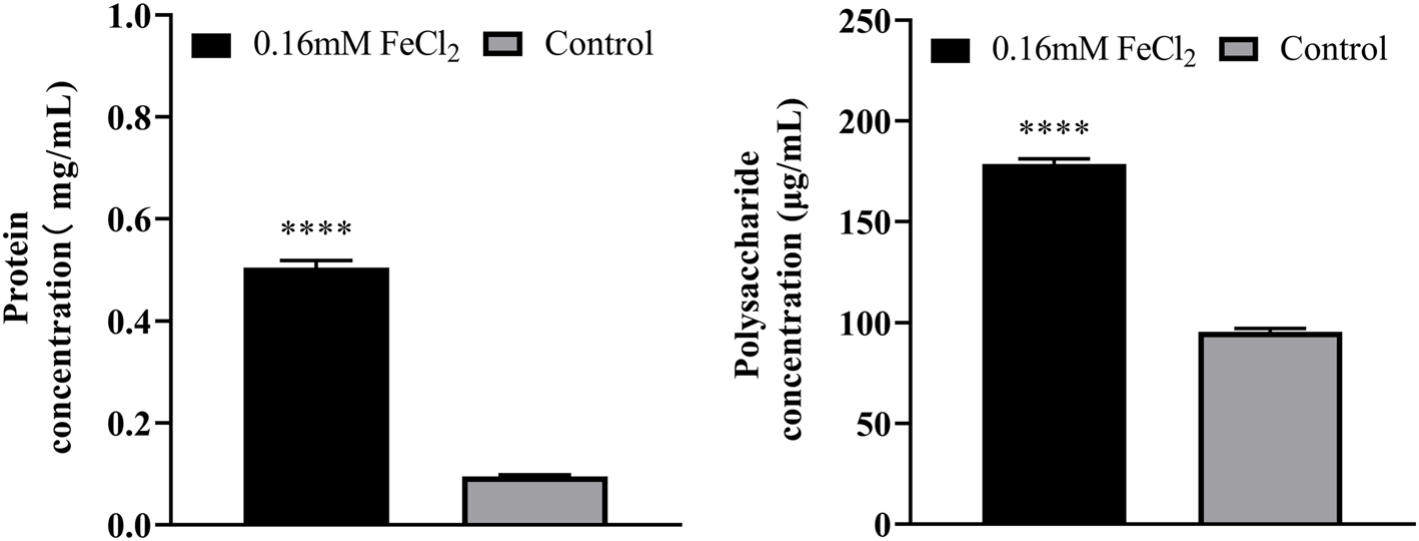
Changes in protein and polysaccharide concentrations in EPS of *K. pneumoniae* YT-9 in the presence of 0.16 mM FeCl_2_. ^****^P < 0.0001 (supplemented vs. control group)

The results of EPS content determination showed that the average protein content in the control group was 0.095 mg/mL, while that in the treatment group was 0.504 mg/mL, which was increased to more than five times. The average polysaccharide content in the control group was 95.6 μg/mL, and that in the treatment group was 178.6 μg/mL, which was also increased to nearly twice. The increase in EPS may be the reason for the more compact and three-dimensional biofilm structure (Fig. 4).

### Metabolomic analysis

**Fig. 5:**
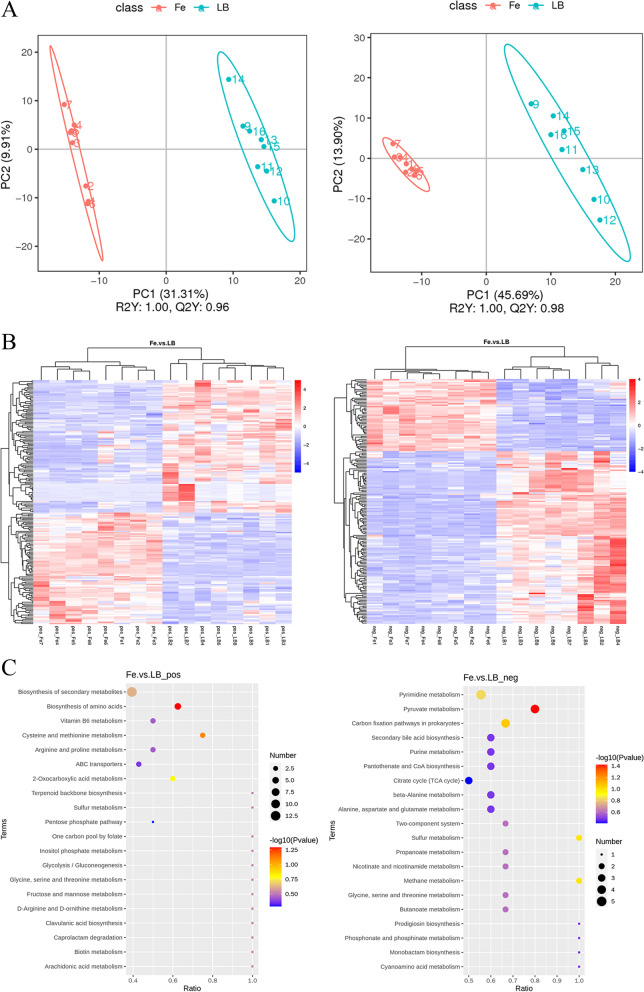
Differences in *K. pneumoniae* YT-9 metabolites between iron-supplemented (Fe) and control (LB) groups based on LC-MS positive ion mode (left) and negative ion mode (right) analyses. **A** PLS-DA of the intracellular metabolome, each data point representing an individual sample. **B** Heatmaps of the differential metabolic production (DMP) in the intracellular metabolome. **C** KEGG analysis of the DMP-enriched biological processes

PLS-DA clearly demonstrated distinct clustering of the groups, indicating that the metabolomic profile of iron-supplemented *K. pneumoniae* was significantly different from the control group (Fig. 5A). Based on the variable importance for prediction (VIP) analysis obtained from PLS-DA modeling and the fold change (FC) and *P*-value data, metabolites with VIP > 1, FC > 5 or FC < 0.667, and *P* < 0.05 were identified as exhibiting significant differences. A total of 361 metabolites differed significantly between the iron supplemented group and the control group, of which 137 increased and 224 decreased. The abundance of significantly differing metabolites was used to cluster the samples and illustrate the relationship between samples and metabolite expression patterns. Fig. 5B shows the hierarchical clustering heatmaps of these differential metabolites in positive and negative ion modes. According to the KEGG enrichment analysis results draw the KEGG enrich scatterplot. Sort the *p*-values obtained by the hypergeometric test from small to large from top to bottom and the smaller the *p* value is, the more significant of the difference. The larger the dot, the more differential metabolites are enriched in the pathway. KEGG analyses indicated that the differential metabolites were mainly associated with enriched pyruvate metabolism and biosynthesis of amino acids (Fig. 5C). This was consistent with previously observed changes in EPS protein and polysaccharide concentrations. Within these two pathways the down-regulated differential metabolites were succinic acid, acetylenedicarboxylic acid, phosphoenolpyruvic acid, acetyl phosphate, O-succinyl-L-homoserine, and N-acetyl-L-glutamic acid. Methionine, S-adenosyl-L-methionine, and L-ornithine were up-regulated.

### Biofilm formation in the presence of succinic acid

This study aimed to identify down-regulated metabolites that could counteract the promoting effects of iron on biofilm formation in chicken water pipelines. Of the six substances that were down-regulated, acetylenedicarboxylic acid is of no practical value because of its strong acidity, while O-succinyl-L-homoserine, phosphoenolpyruvate pyruvic acid, and acetyl phosphate are not suitable for large-scale use because of their high cost. N-Acetyl-L-glutamic acid in the presence of iron promoted the formation of biofilms compared to the control group.

**Fig. 6:**
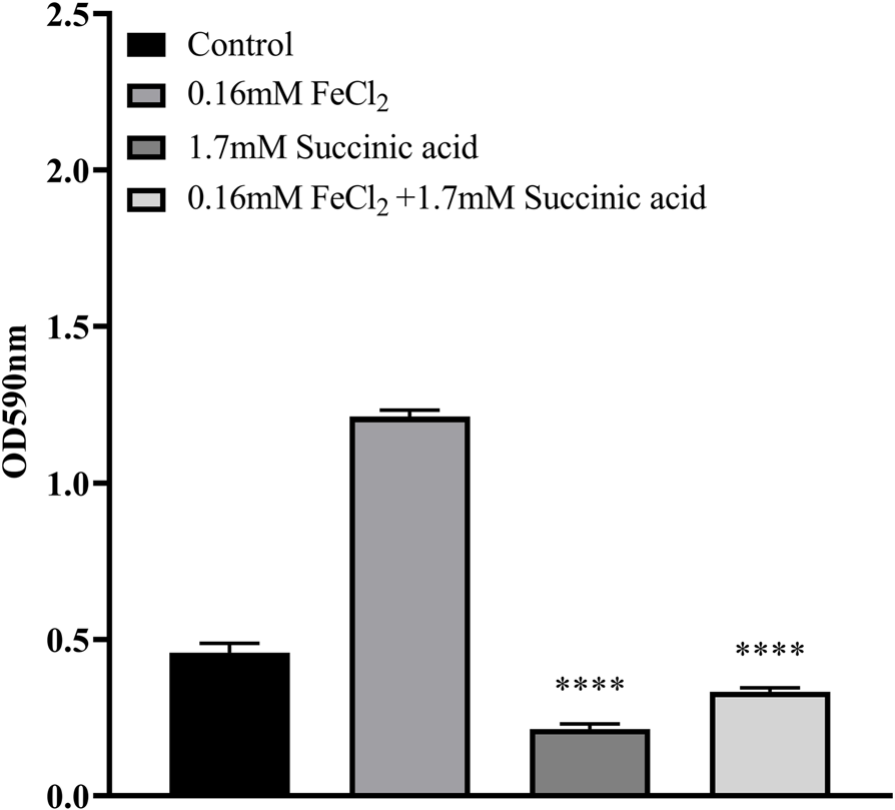
Biofilm formation by *K. pneumoniae* YT-9 in minimal media containing succinic acid or FeCl_2_. ^****^P < 0.0001 vs. 0.16 mM FeCl_2_

**Fig. 7:**
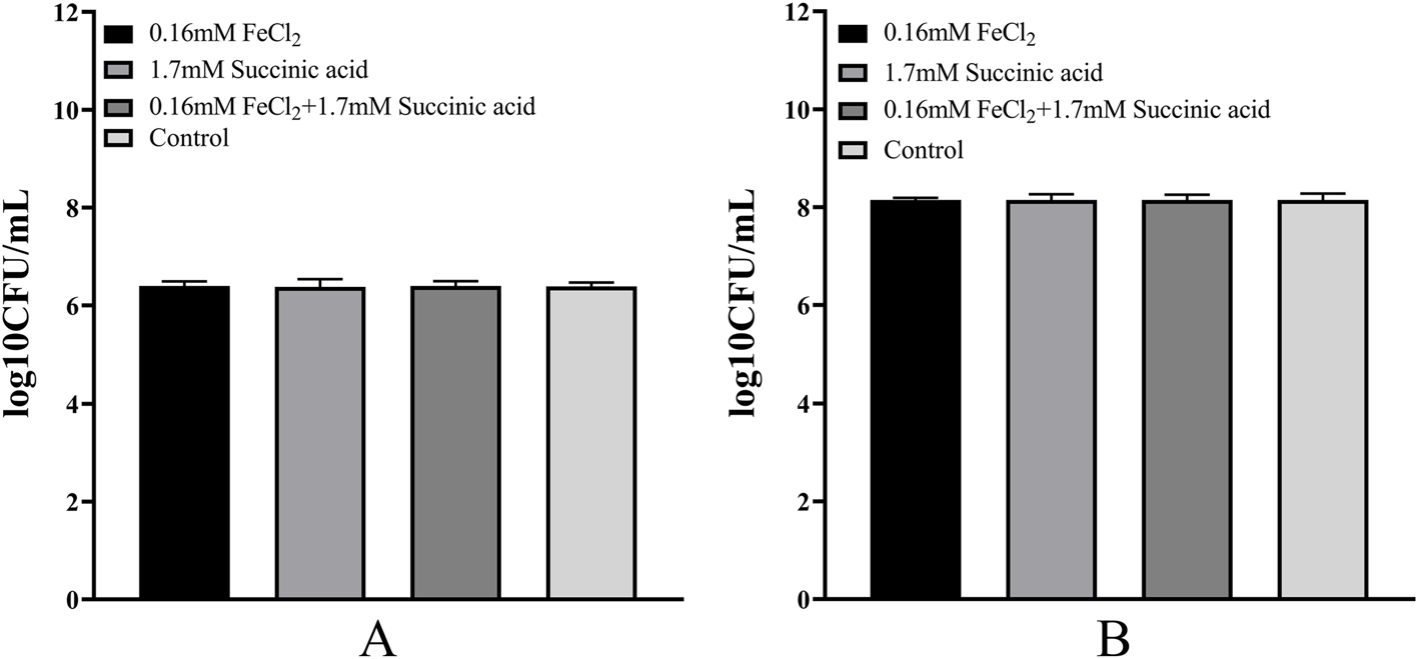
Number of biofilm-forming bacteria (**A**) and planktonic bacteria (**B**) at different FeCl_2_ concentrations and 1.7 mM succinic acid of *K. pneumoniae* YT-9

**Fig. 8:**
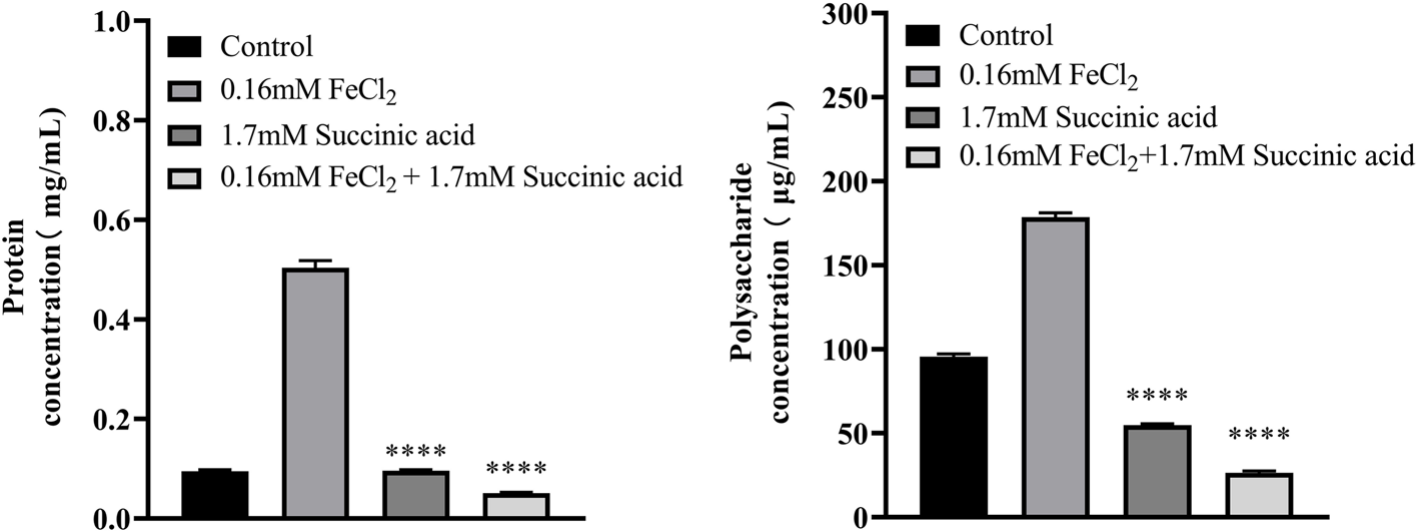
Protein and polysaccharide concentrations in extracellular polymeric substances from *K. pneumoniae* YT-9 biofilm in minimal media containing succinic acid or FeCl_2_. ^****^P < 0.0001 vs. 0.16 mM FeCl_2_

However, the addition of 1.7 mM succinic acid significantly counteracted the enhancing effect of iron on biofilm formation (Fig. 6) and the number of planktonic and biofilm-forming bacteria were not significantly altered (Fig. 7). Analysis of the EPS showed that the protein content decreased to a level similar to the control group when succinic acid accompanied the iron supplementation, while polysaccharide content fell below the control group (Fig. 8).

## Discussion

Biofilms are recalcitrant to antibiotics. Once bacteria form a biofilm, their resistance to antibiotics is greatly increased and a mature biofilm continues to release free bacteria. This makes biofilms a reservoir of drug-resistant bacteria and genes. Due to their moist surfaces, water pipelines are a breeding ground for biofilms, significantly affecting both human and animal piped water supplies. While water pipelines supplying the human population can be regularly replaced and flushed with high-pressure water, such interventions are almost non-existent in the livestock field due to high cost and labor inputs, and livestock drinking water pipelines are often severely affected by biofilms. Iron is essential for the growth of bacteria. Yang et al. [23] demonstrated that *pqs* expression, DNA release, and biofilm formation by *Pseudomonas aeruginosa* were enhanced in media with low iron concentrations (5 μM FeCl_3_). Banin et al. [24] found that limiting iron content reduced *P. aeruginosa* biofilm formation by blocking the early stages of microcolony formation. Chhibber et al. [25] proved that addition of 10 μM FeCl_3_ significantly enhanced biofilm growth by *K. pneumoniae*. Lin et al. [26] found that adding FeSO_4_ to a medium containing an iron chelator restored biofilm formation and production of polysaccharide-intercellular adhesin by *Staphylococcus aureus SA113*.

In this study, low-concentration iron significantly promoted biofilm growth. Of the nine differential metabolites identified by KEGG analysis, six were down-regulated and three were up-regulated. Addition of succinate alongside the iron significantly inhibited biofilm formation. This was possibly due to the succinate participating in amino acid synthesis and pyruvate metabolism, resulting in a decrease in protein and polysaccharides in the biofilm EPS. Metabolomic analysis showed that the concentration of phosphoenolpyruvate decreased in the presence of 0.16 mM iron. Consequently, the downstream products pyruvate and acetyl-CoA also decreased. Acetyl-CoA participates in the tricarboxylic acid cycle, which directly led to a decrease in α-ketoglutarate—the amino receptor for the degradation of almost all amino acids. Without this receptor, amino acids cannot be degraded efficiently, leading to an increase in protein content. Amino acids are precious metabolic fuels. Excess protein metabolism produces amino acids. After deamination, their carbon skeletons can be metabolized to acetyl-CoA, pyruvate, and other tricarboxylic acid cycle intermediates [27], creating a negative feedback process. Succinic acid is downstream of α-ketoglutarate in the tricarboxylic acid cycle, therefore the content of succinic acid was also reduced. Succinic acid is an important part of complex II in the electron transfer chain and its depletion affects efficient electron transfer. Oxidative phosphorylation and electron transport are connected. Electron transfer from the respiratory chain results in the vectorial translocation of protons and this protonic energy is utilized for ATP synthesis [28]. When there is a problem with the electron transport chain oxidative phosphorylation is hindered. Thus, many polysaccharides cannot be degraded and will accumulate outside the cell. From the perspective of material metabolism, the tricarboxylic acid cycle is not only the final pathway of carbohydrate catabolism, but also the pathway for complete oxidation and decomposition of amino acids. When succinic acid is added, the tricarboxylic acid cycle, cell energy supply, and electron transfer are normalized and amino acid degradation and polysaccharide oxidative decomposition return to normal levels, counteracting the effects of the iron. Iron supplementation reduces cellular metabolism and production capacity, causing the bacteria to resemble persister cells. This can be seen as a self-protection mechanism, reducing metabolism to survive in the face of external threats [29].

## Conclusion

Due to the resistance of biofilms to antibiotics it is necessary to develop new antibacterial agents. Iron promotes biofilm formation, but iron-chelating agents are not feasible in agricultural production because livestock and poultry require iron as an essential nutrient. In this study, the addition of succinic acid partially reduced the formation of biofilms by counteracting the effects of iron. Thus, addition of succinic acid to chicken water supplies may be a potential route to reducing biofilm formation in water pipelines. While further exploration and verification by clinical trial are needed, this study provides insight and direction for the development of new antibacterial agents.

## Data Availability

The datasets used and/or analysed during the current study are available from the corresponding author on reasonable request.
